# Dissecting the role of microorganisms in tea production of different fermentation levels: a multifaceted review of their action mechanisms, quality attributes and future perspectives

**DOI:** 10.1007/s11274-023-03701-5

**Published:** 2023-07-29

**Authors:** Matta Assad, Tolulope Joshua Ashaolu, Ibrahim Khalifa, Mostafa H. Baky, Mohamed A. Farag

**Affiliations:** 1 Chemistry Department, School of Sciences and Engineering, The American University, New Cairo, Cairo Egypt; 2 Institute for Global Health Innovations, Duy Tan University, Da Nang, 550000 Vietnam; 3 Faculty of Medicine, Duy Tan University, Da Nang, 550000 Vietnam; 4 Food Technology Department, Faculty of Agriculture, Benha University, Moshtohor, Egypt; 5 Pharmacognosy Department, Faculty of pharmacy, Egyptian Russian University, Badr city, 11829 Cairo Egypt; 6 Pharmacognosy Department, Faculty of Pharmacy, Cairo University, Cairo, Egypt

**Keywords:** *Camellia sinensis*, Tea fermentation, Phenolics, Organoleptic characters, Health benefits

## Abstract

Tea is one of the most popular beverages worldwide, with several health benefits attributed for its rich chemical composition and further associated with fermentation process to improve its quality attributes. Most tea types originate from the leaves of *Camellia sinensis* with differences in fermentation levels yielding black tea, green tea, pouchong tea, oolong tea. Teas like pu-erh or kombucha to encompass both green and red types are further post-fermented. Tea fermentation is a traditional process involving physical, biochemical, and microbial changes which are associated with improved organoleptic characters, nutritive value, and health outcomes. The production of fermented tea relies on naturally occurring enzymes and microbial metabolic activities. This review focuses on presenting a holistic overview on the effect of different microorganisms including bacteria, yeast, and fungi on the biochemical changes and sensory attributes of fermented tea products reported in research articles along the last 15 years. Moreover, production conditions and major biochemical changes are dissected to present the best factors influencing fermented tea quality. This review presents an evidence-based reference for specialists in tea industry to optimize tea fermentation process for targeted attributes.

**Graphical abstract:**

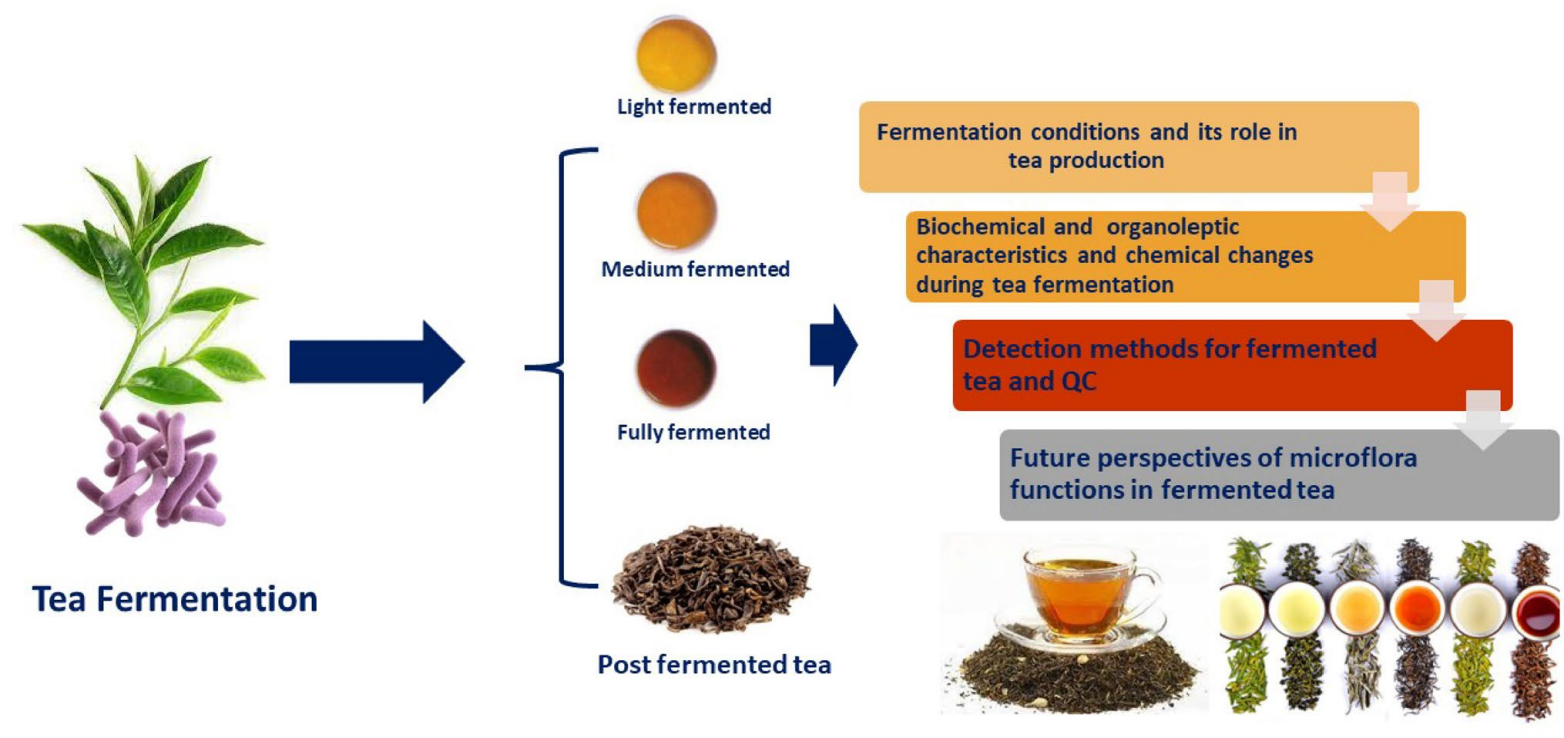

## Introduction to the different fermented tea and their health benefits

Tea (*Camellia sinensis*) is the source of the second most consumed beverage worldwide after water (Pasrija et al. 2015; Mandal et al. 2022). Owing to its richness in several active and functional molecules, tea plays a pivotal role in human health. The different tea types are classified according to the degree of fermentation including green tea where no fermentation or oxidation occurs, white tea is a light fermented, oolong tea is a semi-fermented, and black tea is fully fermented and oxidized (Jolvis Pou 2016). Moreover, Pouchong tea is only 8–15% oxidized and considered as the finest tea in the world and falls between green tea and oolong tea (Chiang et al. 2020). Pu-erh, Qingzhuan, and Fu-brick are all examples of post-fermented teas (Xu et al. 2015). Compared to other tea types, Chinese dark teas, and post-fermented teas require special microbial processing called the “pile process” which includes both non-enzymatic autoxidation and enzymatic oxidation to finalize the characteristic taste, color, and aroma of such tea products (Lu et al. 2016).

Tea fermentation process is considered as the driving key method responsible for the different biochemical changes and production of bioactive molecules leading to improvement of tea sensory attributes (Vahabzadeh et al. 2009). Such endogenous phenomenon mostly targets enzyme-mediated oxidation of tea polyphenols (Ghosh et al. 2015). As a general procedure, the freshly collected tea leaves are subjected to heat treatment to deactivate endogenous enzymes and elongate the storage period. After drying, an adequate amount of water is added to the product before allowing natural fermentation to take place. The fermentative strains act on the tea leaves leading to color and flavor changes, and are regularly monitored by an experienced operator to determine the endpoint of the fermentation process (Abe et al. 2008).

The type of microbial species can contribute to tea quality. For instance, *Eurotium cristatum* (*E. cristatum*) that causes the conversion of phenolics initially present in unfermented green tea adds certain desirable features of taste, color, and flavor to the fermented tea product (Rui et al. 2019; Xiao et al. 2020). The involved microorganisms not only produce functional enzymes, but rather secrete active ingredients that provide tea its unique characteristics, functionality and ultimately health benefits (Zhu et al. 2020). Probiotic microbial strains have been shown to exert several local health effects to the gastrointestinal tract as well as systemic actions. For example, *Bacillus coagulans* (*B. coagulans*) is a safe thermoduric probiotic used in food products such as natto (traditional Japanese fermented soybeans) to improve taste and shelf life (Endres et al. 2009). This probiotic bacterium is used in the fermentation of pu-erh tea to improve its health benefits (Zhao et al. 2013). Identifying such microbial species requires on top microbiological techniques for isolating targeted bacteria such as culture-based and sequencing-based techniques. Recently, DNA sequencing of the species are used as the base of detection and aided by epigenetic sequencing approaches (Marsh et al. 2014; Unban et al. 2020).

Fitting the consumer satisfaction and meeting the different preferences are the main goals in developing tea production industry. Ensuring tea quality is considered of utmost value and monitored by several components such as theobromines and polyphenols. which affect sensory characteristics of the produced beverage (Xie et al. 2009). Each tea has its own characteristics, for example, pu-erh shucha exhibits a red-brown liquor with a mellow stale flavor attributed to the fermentation process. Recently, there is a continuous growth in consumers´ interest in fermented food products owing to their health benefits. Kombucha tea produced by fermenting tea and sugar with bacteria and yeast strains (Teoh et al. 2004) was reported to exhibit anti-diabetic (Aloulou et al. 2012), antimicrobial, antioxidant (Bhattacharya et al. 2013) and anti-carcinogenic effects (Jayabalan et al. 2011). Moreover, kombucha tea showed positive effect in gastric ulcers treatment (Banerjee et al. 2010), decrease cholesterol, boost immune response, and several other health benefits (Vahabzadeh et al. 2009). Another health promoting tea is fuzhuan brick tea that comes from the old, rough and coarse *C. sinensis* leaves, and known to act as a hypolipidemic, antiproliferative and anti-obesity beverage in Japan, Korea and China (Xiao et al. 2020).

However there are several reports on fermented products (Kosseva and Technology 2011), only solo fermented tea products have been investigated such as yellow tea (Horžić et al. 2012), green tea (Pasrija et al. 2015), fresh tea leaves (Liu et al. 2019), and Kombucha tea (Ramírez Tapias et al. 2020). Hence, this multifaceted review aims to dissect for the first time all issues related to the role of microorganisms and microbial enzymes in fermented tea production, starting from culture classes, reaction mechanisms underlying fermentation process and quality attributes. In addition to an objective compilation of factors affecting fermentation process and detection methods to ensure best tea quality.

## Tea fermentation impact on biochemical characteristics and chemical composition

**Fig. 1:**
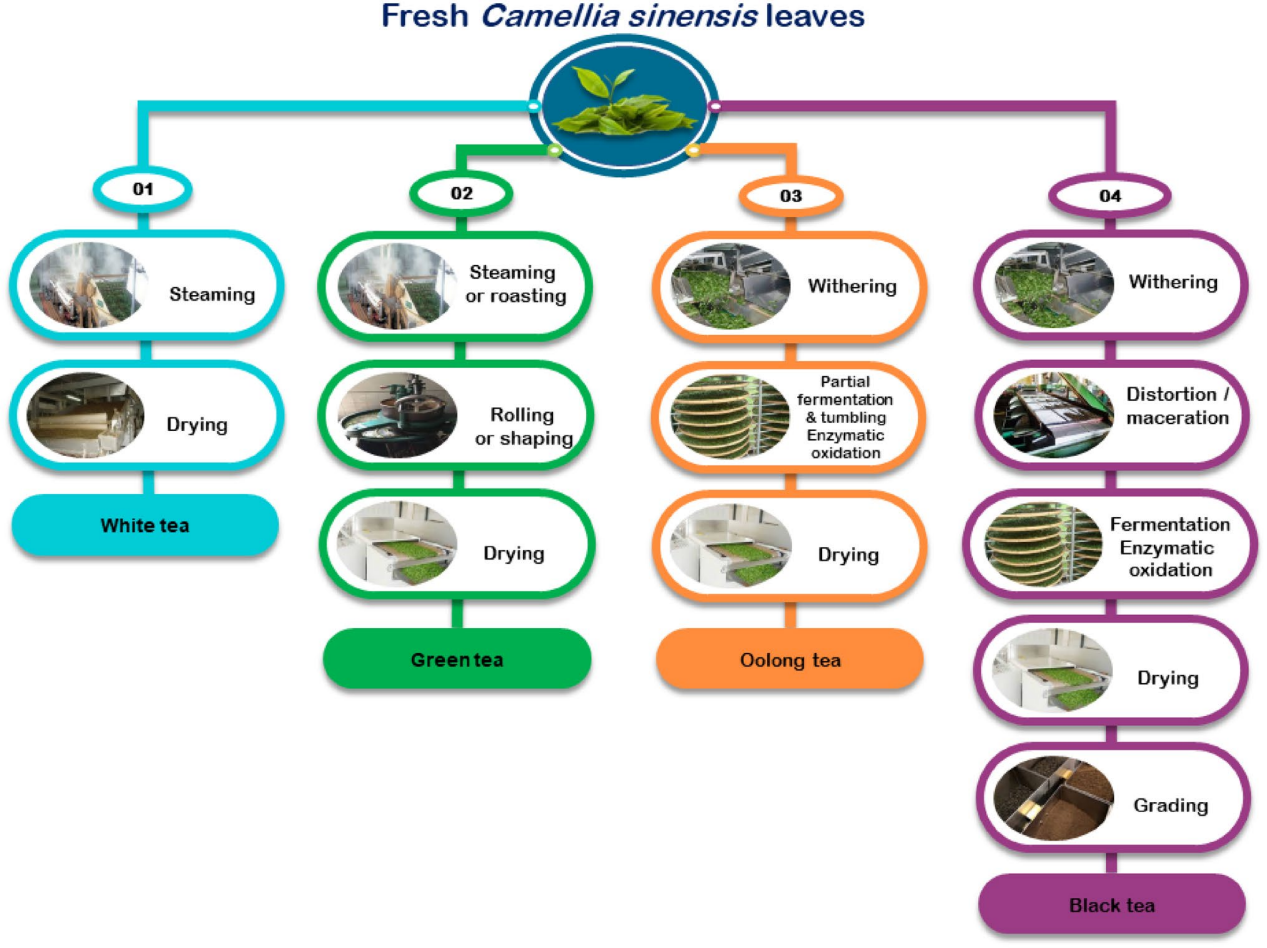
The tea fermentation steps from *C. sinesnis* leaves to yield different tea products in the market

Tea processing is an overlong procedure with many steps including plucking (picking), withering, rolling, fermentation, drying, and sieving. The detailed tea fermentation steps are described in Fig. 1.

During tea processing, a myriad of biochemical transformations occurs during the withering phase, including the breakdown of complex chemical compounds into simple volatile ones (amino acids and simple sugars), degradation of chlorophyll, and elevation of enzymatic activity (Deb and Jolvis Pou 2016). Although weathering is responsible for many biochemical changes, the most discernible changes occur during the fermentation step. Tea fermentation is a mechanistic process by which polyphenols located in tea leaves get oxidized through either endogenous enzymatic polyphenol oxidase or microorganisms, including yeasts, bacteria, and fungi (Zhang et al. 2019) as outlined in the next section.

Fermentation is vital for the improvement of tea aroma, color, taste, quality, and mellowness (Samanta et al. 2015). The flavor development occurs by altering tea leaves´ chemistry, resulting in a change in their organoleptic qualities (Zhong‐Yi et al. 2010). Various tea types are processed and manufactured by different ways of fermentation which result in subsequent biochemical variation among their polyphenolic content. The major polyphenols found in tea include catechins, flavins in addition to sugars, proteins, and alkaloids which vary according to different tea types as illustrated in Table 1. Fully fermented and post-fermented teas, such as black and pu-erh teas are enriched in theaflavins (TFs) and thearubigins (TRs), compared to white, green, and yellow teas which have lower TFs and TRs levels and higher catechins and flavins (Rahman et al. 2020). Owing to fermentation, the oxidized polyphenols such as TFs and TRs are responsible fora stronger taste, odor, and color whenever it is exposed to a longer period of fermentation. Pu-erh tea has a very strong flavor when further processed after a complete fermentation process. The major sensory characteristics controlling tea products’ quality include flavor, taste, aroma, and color which are strongly dependent on the fermentation period. Thus, the next subsections highlight how fermentation affects tea organoleptic characters, chemical composition as well as optimization to improve each sensory attribute.

**Table 1:** List of chemical compounds responsible for flavor, odor, and taste of the different fermented tea types

Tea type	Level of fermentation	Tea properties	Compounds responsible for:			References
			Aroma	Taste	Color	
Black tea and pu-erh tea	Black tea is a fully fermented tea, Pu-erh tea is a post-fermented tea	Sweet, grassy, fruity, and floral, It has a citrus, fresh, and minty scent, Rough and ashy appearance, Bitter and slightly astringent tastes	β-Damascenone, followed by trimethoxybenzene, (*E,E*)-2,4-nonadienal, linalool, hexanal, phenyl, ethanol, γ-terpinene, linalool, 4-ethylveratrol	Methyl salicylate, caffeine, catechin, epigallocatechin gallate, benzaldehyde, and amino acids	Theaflavins, thearubigins	Bhattacharyya et al. (2007), Chaturvedula (2011), Chen et al. (2013), Stodt et al. (2014), and Zhao et al. (2018)
Yellow tea	Light-fermented Tea	Cleaner and fresher, aged, fungal, aroma, mellower and less bitter without the grassy taste of green tea	Catechin, epicatechin, gallocatechin, epigallocatechin	Catechin, epicatechin, gallocatechin, epigallocatechin, gallocatechin-gallate, epigallocatechin-gallate, epicatechin-3-gallate	Kaempferol, quercetin, isoquercetin, myricetin, apigenin, vitexin, vicenin-2, and glycerides	Xu et al. (2018a, b)
Green tea	Non- fermented tea	Bitter and grassy, raw smell	Hydrocarbons, alcohols, acyclic monoterpenoid (linalool), aldehydes, ketones, esters, and phenols	Aspartic and glutamic acids	Chlorophyll a and b.	Wang et al. (2004)
White tea	Very light-fermented Tea	Sweet	–	Flavonoids, glutamate and other amino acids	–	Horanni, Engelhardt, & Analysis, (2013)

## Fermentation and withering processes impact on tea aroma

Withering as a first fermentation step has a significant impact on final product sensory attributes as it induces certain biochemical interactions that play a pivotal role in product aroma (Soheili-Fard et al. 2015). Prior to any fermentation steps, the fresh green tea leaf is composed of 30% catechins while other compounds belong to other classes such as flavanols, terpenes, and phenols (Liu et al. 2019). Of these chemicals, linalool and hexanal, 1-penten-3-ol, 2-penten-1-ol, benzaldehyde, *β*-damascenone, (*Z*)-3-hexenylhexanoate and (*Z*)-3-hexenyl-2-hexenoate were key aroma compounds in green tea (Guo et al. 2021). As the tea fermentation proceeds, a fruity aroma is developed, which diminishes over time. Amino acids in tea can combine with orthoquinone (oxidized form of catechin) and generate the characteristic tea aroma (Jolvis Pou 2016). Thereafter, a more unique fruity aroma appears with the formation of the coppery brown color, indicating the end of the fermentation process (Jolvis Pou 2016; Sharma and Rao 2009).

With regards to extended post-fermented teas, such as pu-erh, more than 630 aroma compounds have been identified belonging to various aliphatics, alicyclics and aromatics (Chaturvedula and Prakash 2011). Among these aroma compounds, *β*-damascenone, followed by 1,2,3-trimethoxybenzene and (*E, E*)-2,4-nonadienal were potential markers that contributed to the aroma of pu-erh tea. Other volatiles such as γ-terpinene, linalool, 1,2,4-trimethoxybenzene, 1,2,3-trimethoxybenzene, and 4-ethylveratrol were also identified as contributors to aroma differences in pu-erh tea fermentation stages (Deng et al. 2021). Aside from terpenes, the distinguishable, unique aroma of fermented tea has been linked more specifically towards the decomposition of lipids and carotenoids. Carotenoids are known to be subjected to oxidation reactions yielding aromatic compounds, whereas glucosidases hydrolyze glycosidic-bound aroma compounds yielding stronger aroma in fermented tea.

Several factors determine the number of volatiles present in tea. For instance, Zhao et al. (2018) concluded that the longer the duration of kombucha tea fermentation, the lower the degradation of catechins and caffeine, the lower the pH, and the higher the number of volatile compounds. Among these volatiles, alcohols were the largest group detected in kombucha tea (Zhao et al. 2018). Furthermore, amino acids play an important role in determining tea aroma. During fermentation, amino acids merge with orthoquinone, which is an oxidized structure of catechin, initiating the formation of several volatile compounds found in the black tea aroma fraction (Pasrija et al. 2015). Thus, carotenoids, amino acids, and glucosides largely contribute to fermented tea aroma through hydrolysis and oxidation reactions to act as aroma precursors. Consequently, profiling of these non-volatile precursors in green tea accessions can provide hints of the best aroma characteristics to be observed upon fermentation.

## Fermentation and withering processes impact on tea taste

Besides fermentation process, there are other factors that determine tea taste, including astringency, bitterness, umami, sweet after taste, floral flavor, etc. In green tea, astringency has been associated with the presence of flavonoids (Xu et al. 2018b), whereas compounds such as non-galloylated epigallocatechin and epicatechin have been recognized as precursors of a sweet aftertaste (Cao et al. 2019; Zhang et al. 2016). Regarding tea bitterness, catechins (flavonoids) are one of the major compounds that are responsible for its bitter taste (Narukawa et al. 2010). These flavor-associated compounds were found to change at different times during the semi-fermentation of oolong tea (Liu et al. 2018). Furthermore, esters e.g., ethyl salicylate, and alkaloids e.g., caffeine showed increase throughout the fermentation process concurrent with a decrease in aldehydes (Liu et al. 2018; Li et al. 2018b; Qin et al. 2012).

Caffeine as an alkaloid is known to impart bitter taste to tea products (Zhang et al. 2020). Thus, the more prolonged the fermentation process, the higher the caffeine level, and the bitter the tea product. Aside from esters, alkaloids, and flavonoids, umami amino acids that are responsible for the sweet flavor of the Japanese green tea were found to be associated with the nonprotein amino acid, theanine (Pasrija et al. 2015).

Furthermore, during tea fermentation such as in kombucha, *Acetobacter sp.* and *Acetobacter aceti* were the main microbes producing organic acids, such as acetic acids, starting from the 5th day of fermentation (Zhao et al. 2018). This indicates that the choice of microbes included during the fermentation process does not only affect polyphenolics level, but rather affect other compounds such as acids thereby imparting a different taste to the tea. Overall, through the complex biochemical alteration of polyphenols, fermentation affects not only tea color, but it also typically mellows its taste, reducing astringency and bitterness while improving mouthfeel and aftertaste (Cao et al. 2019). Hence, fermentation and withering processes have a great impact on tea product quality by enhancing the sensory attributes including taste and flavor through enhancing several key biochemical reactions.

## Fermentation and withering processes impact on tea color

The variations in the pre- and post-fermentation processes play a role in the discrepancy of biochemical changes accounting for tea effects as well as the colors of different tea types. Different colors of tea have been viewed as a reflection mostly for change in polyphenols (Pou 2016). Compared to other tea types, white tea exhibits a very light fermentation phase as it undergoes decolorization and drying (Tan et al. 2017). During tea decolorization, amino acids pool was shown to be inversely proportional with tea fermentation; hence, free amino acids such as γ-aminobutyric acid (GABA) and asparagine showed elevated level in white tea (Horanni and Engelhardt 2013).

On the other hand, in terms of colored teas, green tea chlorophyll pool proved to be the prominent compounds for dry tea leaves color through the ratio between chlorophyll a and chlorophyll b. During green tea infusion, water-insoluble chlorophylls are released from the fragile tea leaves, leading to the high greenness and turbidity (Pasrija et al. 2015). In addition to chlorophylls, quercetin, a flavonol, was shown to be the chief phenolic contributing to the greenness of tea infusions (Pasrija et al. 2015). Although yellow tea is processed with the same technology used for green tea, the yellowness of tea leaves is produced through a technique called “sealed yellowing”, in which tea is encased and steamed to allow for oxidation to occur at a slower rate, converting its green color into yellow and changing tea quality (Xu et al. 2018b). The yellow color has been associated with soluble flavanols (kaempferol, quercetin, isoquercetin, and myricetin), flavones (apigenin, vitexin, and vicenin-2), alongside their glycosides (Chaturvedula and Prakash 2011).

When it comes to the unique color of black tea, it has great appeal for consumers due to its texture, sensory features, health-related benefits, and appearance. The black color develops upon catechins condensation aided by enzymatic oxidation to form large molecules of nonvolatile TRs and TFs (Bhattacharyya et al. 2007). An example of such phenolics oxidation is the low content of catechins in pu-erh tea due to its complete oxidation, which gives it its silky, smooth, and mellow appearance as well as its reddish-brown to dark black color (Chen et al. 2013). Both TFs and TRs content could be used as a reference measure for the quality of tea liquor, taste, and color. TFs and TRs are also accountable for the shading of black tea, including the orange-red and reddish-brown colors of the tea (Stodt et al. 2014). With regards to the color of pu-erh tea, the use of microbial fermentation as adjuvant such as *S. bacillaris or S. cinereus* culture were found to enhance the pu-erh tea color during the fermentation process (Wang et al. 2015).

## Fermentation conditions and their role in tea production

**Fig. 2:**
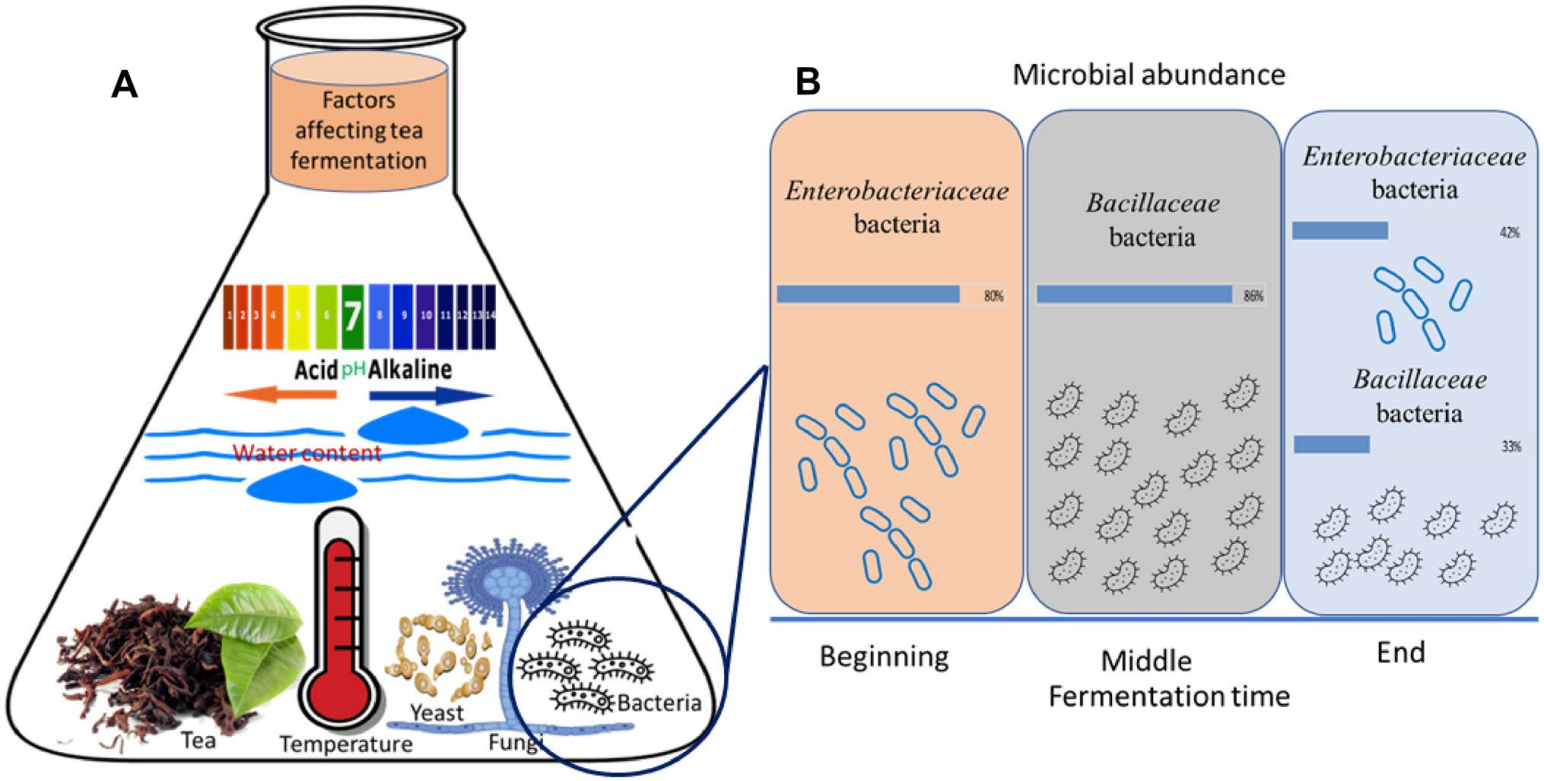
** A** Diagrammatic sketch depicting key factors involved in tea fermentation process represented in pH, water content, microorganisms, and temperature. **B** The microbial abundance of two main bacterial families throughout the fermentation process of pu-erh shucha tea

This section discusses the most common prevalent bacterial, yeast and fungal organisms employed in the different types of fermented teas with some possible linked metabolites, growing conditions of temperature and water content and metabolism that could be optimized for the best fermentation conditions in tea (Fig. 2) Table 2.

**Table 2:** List of the most commonly isolated microbes in fermented teas

Tea type		Microbe		Microbial contribution/characteristics	References
Name	Fermentation type	Name	Type		
kombucha	black tea	*Acetobacter xylinum*	Bacteria	Acetic Acid Bacteria Difficulty to isolate with culture method	De Filippis et al. (2018)
		*G. xylinus*			
		*Lactobacillus*	Yeast	Sugars metabolism Grows at 1.5 pH, 37 °C, 40% sucrose, and 2% bile and survive both gastric and intestinal simulated conditions	Tu et al. (2020)
		*Starmerella davenportii* Do18			
Xiaguan Tuo	pile-fermentation (SSF)	*Bacillus*	Bacteria	Non-spore bacteria isolated by culture-dependent approach	Li et al. (2018b)
		*Coccus*			
		*A*. *niger*	Fungi	Develop aroma compounds	Abe et al. (2008; Li et al. (2018a)
		*B*. *adeninivorans*		Secretion of enzymes	Li et al. (2018a)
		*Penicillium*		Antibacterial (safer tea)	
		*Rhizopus*			
Pu-erh	SSF	*Klebsiella*	Bacteria	88% of total bacteria	Li et al. (2018b)
		*Lactobacillus*	Fungi	Provides mellow taste, stale flavor, and red-brownish red liquor of the fermented tea	Zhao et al. (2013)
		*Actinoplanes*, *Streptomyces*, *Paenibacillus* *Bacillus*			
		*A*. *niger*		Develop aroma compounds	Abe et al. (2008)
		*B*. *adeninivorans*			
		*Aspergillus sydowii*		Conversion of caffeine into theophylline	Zhou et al. (2019)
		*R. emersonii* *R. pusillus* *A. fumigatus* *A. niger*		Higher temperature with acidic parameters for optimal growth	Zhang et al. (2016)
	Submerged pure culture fermentation	*Rhizomucor pusillus, Aspergillus tubingensis, Aspergillus fumigatus, Aspergillus marvanovae, Rhizomucor tauricus*		Converting polyphenols into TB in higher productivity compared to SSF	Wang et al. (2015)
Miang	Not mentioned	*Lactobacillus*	Bacteria	LAB, culture-independent	Unban et al. (2020)
		*Acetobacter*			
QZBT	SSF	*Bacillus subtilis*		Enzymatic release Isolated at 45 °C	Xu et al. (2019)
		*Bacillus oryzaecorticis*			
Fu-brick	Liquid-state fermentation	*Eurotium cristatum*	Fungi	Potential probiotic with several health benefits Instant dark tea aroma	Chen et al. (2021); Lu et al. (2022)
		*A*. *niger*		Decomposition of cellulose and hemicellulose Higher TB level Instant dark tea aroma	Chen et al. (2021)
Fuzhuan brick	Not mentioned	*Eurotium cristatum*		Presence of anthraquinones	Xu et al. (2011)
		*Aspergillus*		Other fungal isolates	
		*Pestalotiopsis*			
		*Rhizomucor*			
		*Verticillium*			
		*Beauveria*		Impart protection against insect tea spoilage	
Dark fermented tea	Not mentioned	*Aspergillus egyptiacus*		Marker for tea quality	Xu et al. (2018c)

### Bacterial cultures

Kombucha tea is a slightly acidic and carbonated beverage formed by sweetened tea fermentation with addition of a symbiotic consortium of bacteria and yeast(Coelho et al. 2020). As a part of the consortium involved in kombucha, bacterial inoculum ferments the sweetened tea. After a period of 8–10 days, acetic acid, ethanol, and CO_2_ are typically produced. As the dominant bacteria, acetic acid bacteria (AAB) are utilized in kombucha tea to drive fermentation. Alongside osmophilic yeasts, AAB develops a floating biofilm of cellulosic pellicle on the fermented medium, while they stay embedded inside the liquid. Dominant species such as *Acetobacter*, and other species of *Gluconacetobacte*r and *Lactobacillus* were isolated from fermented kombucha although with the difficulty of isolating AAB using culture method (Trovatti et al. 2011). As a cellulose producer in the formed floating pellicle, *Acetobacter xylinum* is the best species among AAB. In liquid media, fermentation increased the colony forming units (CFU) number by 1.5 log for AAB which has led to a rapid decrease in pH from 3.5 to 2.5 after a week. Variables such as fermentation temperatures of 20 and 30 °C and tea types (green or black) showed no significant impact on the fermentative AAB when two different growth media were used (De Filippis et al. 2018).

Regarding bacterial diversity, fermentation temperatures of 20 and 30 °C when applied for a day expressed higher diversity as 80% *Gluconacetobacter* and 10% *Acetobacter* were detected at 20 and 30 °C fermentation temperature, respectively. However, after 21 days, change in abundance was observed for both genera at both temperatures manifested by 10% increase in *Gluconacetobacter* and decrease to less than 5% in *Acetobacter* population. The higher temperature of 30 °C favored the growth of several bacteria such as *Lactobacillus*, *Streptococcus*, *Lactococcus*, *Propionibacterium* and *Corynebacterium*. Additionally, higher temperature affected *Gluconacetobacter xylinus* (*G. xylinus*) (prevailed at 20 °C) and *G. saccharivorans*, (boosted at higher temperature) (De Filippis et al. 2018). Such pattern can support the development of temperature-organism prevalence-oriented programs of incubation so that the growth or inhibition of certain bacteria could be controlled.

In the post-fermented black tea, Xiaguan Tuo tea, bacterial contribution had little impact of ca. 10^3^ cells/g during tea fermentation compared to fungi and yeast. During its pile-fermentation step, non-spore *Bacillus* and *Coccus* bacterial communities were isolated by utilizing a culture-dependent approach. Pile-fermentation is the main step in the production of dark tea and is considered solid-state fermentation (SSF). During this step, characteristic features of taste, flavor and color of dark tea are generated by the effect of changes over time alongside growth of microbial communities (Li et al. 2018b).

Due to the limitation of culture-dependent isolation techniques to identify some strains, other culture-independent methods are typically employed in which no culture step is involved such as denaturing gradient gel electrophoresis (DGGE), terminal restriction fragment length polymorphism (T-RELP) and high-throughput sequencing technology to identify microbial composition of fermented teas. With such methods, *Bacillus* and *Enterobacteriaceae* genera, for example, were identified from pu-erh tea. As dominant genera during pile-fermentation of dark tea, *Klebsiella* and *Lactobacillus* accounted for more than 88% of the microbial consortium. Moreover, several functional core microorganisms, including genera *Aspergillus*, *Bacillus, Candida, Cyberlindnera, Debaryomyces, Eurotium, Klebsiella, Lactobacillus*, and *Lactococcus* are included in dark tea fermentation and production of functional components (Lin et al. 2021). Monitoring changes in bacterial genera showed dramatic decrease of *Lactobacillus* from 7% at the initial stage to reach 0.5% after 12 h, concurrent with the domination of *Klebsiella* during the whole process. However, other bacterial genera detected at less than 3% included *Kluyvera*, *Methylobacterium*, and *Aurantimonas* (Li et al. 2018b). In Thai tea also known as “miang”, lactic acid bacteria (LAB) dominated microbial consortium followed by *Bacillus* genus with changes over time. There appeared to be an antagonistic effect between LAB and yeast that aided to stabilize the whole fermentation process (Unban et al. 2020). This could be associated with the different metabolic patterns exhibited when competing for the same substrate for microbes’ viability and growth.

Using culture-independent high-throughput sequencing method, two dominant bacterial phyla were identified belonging to *Firmicutes* and *Proteobacteria*. 11 Bacterial families were recognized dominated by *Lactobacillaceae* (40–80%), with *Lactobacillus* accounting for 30–77% of the population, while *Acetobacter* represented 4–23% of the family (Unban et al. 2020).

LAB can modify pH of teas by increasing total acids level. LAB growth in Miang tea was accompanied by increased lactic acid level at the beginning of the fermentation compared to other acids reaching 51 mg/g on the 9th day followed by butyric acid predominating after a month of fermentation. *Clostridium*, an anaerobic spore-forming bacteria oxidizes sugars to pyruvate, and then to butyric acid. Its growth depends on a decrease in oxygen level which normally occurs during the mid-time of fermentation (Unban et al. 2020).

Pile-fermentation is considered the most critical step in tea production as demonstrated in the production of Qingzhuan brick tea (QZBT). The fermentation is initiated upon the inclusion of certain microbial communities to tea leaves leading to the production of various dark tea chemical features such as enzymes production. To elaborate, *Bacillus subtilis* and *B. oryzaecorticis* with other microbes contribute to the release of pectase, protease, cellulase and polyphenol oxidase. *Bacillus* also augmented the bioconversion capacities of other species to develop QZBT’s biochemical characteristics. For example, *B. subtilis* combined with other species shortened the period of pile-fermentation (Xu et al. 2019).

Culture-independent detection methods have some limitations involved in the identification of the microbial communities that grow during tea fermentation. The higher stability of DNA of the sample under detection can be misleading as it cannot reflect the actual prevalent conditions of living structures, with dynamic changes that occur in microbial strains during pile-fermentation due to an increase in temperature. Such increased thermal changes can stop the growth of certain strains, whereas other thermoduric strains could grow and rapidly multiply (Xu et al. 2019; Junlin et al. 2017).

Novel closely related Gram-positive bacteria i.e., *Isoptericola cucumis* from *Promicromono sporaceae* family was recently isolated from pu-erh tea in a pile-fermentation process suggestive for more studies to completely map out the composing microbial communities using different approaches. This will enable better understanding of their contributions towards color, taste, aroma, and active molecules in fermented tea. Identification of these fermentation-involved microbes in different teas, alongside well organized databases can be attained leading to improved fermentation processes and tea attributes (Yang et al. 2021).

The microbial content of pu-erh shucha contributes to its characteristic properties such as the mellow taste, stale flavor, and reddish-brownish red liquor. Examples of bacteria isolated from this tea include *Actinoplanes*, *Streptomyces* (Chen et al. 2010), *Paenibacillus* (Kim et al. 2009), and *Bacillus* (Miao 2011). In a different study conducted on pu-erh shucha, there was a change in the dominating bacteria during fermentation time. At the beginning and until the 10th day, bacteria belonging to the *Enterobacteriaceae* family represented more than 80% of the bacterial population, while the balance shifted towards *Bacillaceae* on the 15th day dominating 86% of the population. The high loads of *Enterobacteriaceae* of the starting material should check for any possible pathogenicity such as *Salmonella*, *Pseudomonas*, *Yersinia*, and *Vibrio* strains belonging to this family. Upon analyzing the isolated *Bacillaceae*, the dominant strains showed a high similarity with the thermoduric *B. coagulans*, which exerts probiotic effects (Zhao et al. 2013).

The average temperature in pu-erh shucha fermentation ranges between 40 °C and 60 °C as it fluctuated during the process. With such change, abundance of bacterial diversity also changes. For example, *B. coagulans* showed a higher dominance (86%) upon temperature increase from 40 °C to 60 °C. Collective data monitoring the abundance of strains at corresponding temperatures could provide a better insight on the fermentation process dynamics and aid future optimization targeting abundance of certain bacterial strains for a desired outcomes in fermented tea products (Zhao et al. 2013). Recently, Zhu et al. studied the effect of thermophilic microorganism pile-fermentation on Chinese dark tea production revealing reduction of (−)-epigallocatechin gallate, (−)-epigallocatechin, (−)-epicatechin gallate, and (−)-epicatechin levels. Moreover, thermophilic microorganism pile-fermentation significantly influenced caffeine metabolism and increased the level of theophylline, 3-methylxanthine, and 1,3,7-trimethyluric acid (Zhu et al. 2022).

### Yeast cultures

Bacteria is not the only organism involved in tea fermentation; yeasts likewise play a role in this process. For example, isolated *Zygosaccharomyces sp*. from kombucha tea played a role in the breakdown of sugars i.e., production of fructose and glucose from sucrose. Alongside other microbes, yeasts such as *Saccharomyces*, *Pichia*, *Schizosaccharomyces*, *Brettanomyces*, *Saccharomycodes*, *Candida* and *Torulaspora* were all isolated from several fermented black teas. Interestingly, these yeast microbes were unequally distributed on and within the formed pellicles in growth cultures, where tea fermentation occurs (Li et al. 2018a).

The conversion rate of glucose and fructose to CO_2_ and ethanol relies on the type of yeast cells and their metabolic activities, with fructose fermentation by yeast cells to generally occur at a higher rate than glucose. Thus, the ratio between fungal species and yeast cells participating in tea fermentation need to be optimized to drive the metabolic pathway into a specific direction at targeting certain output for desired tea attribute (Aung et al. 2022).

Microbial activities that occur during tea fermentation could influence different chemical compounds’ profiles from sugars to alkaloids, polyphenols, and terpenes, which then influence tea health benefits. Tannins and caffeine as the major bioactives in tea, for instance, exhibited a significant decrease, which is concurrent with a 100 folds increase in theophylline level in fermented black tea through the metabolic action of *Debaryomyces hansenii* (Wang et al. 2020). Black tea fermentation by *Dabaryomyces hansenii* results in the reduction of caffeine and amount of tannins levels and improves both nutritional and health value(Hu et al. 2022).

Some of these compounds could be volatile, thus contributing to tea aroma composition and specificity of flavors upon yeast fermentation. For example, *Saccharomyces cerevisiae* (*S. cerevisiae*) can play a crucial role in the isomerization of geraniol into linalool and α-terpineol, whereas *Kluyveromyces lactis* and *Torulaspora delbrueckii* catalyze the conversion of geraniol to its isomer citronellol (Wang et al. 2020).

Other non-saccharomyces yeasts less explored for their potential in tea fermentation include *Pichia kluyveri Frootzen* (*P. kluyveri*), *Torulaspora delbrueckii Prelude*, *Torulaspora delbrueckii Biovada* and *Williopsis saturnus var. mrakii* NCYC2251 (*W. saturnus*). Initial studies showed that their fermentative power could degrade flavor precursors (*P. kluyveri*), convert alcohols into esters (*W. saturnus*), and produce terpenes and fruity esters (*Torulaspora delbrueckii*) in products other than tea that has yet to be exploited in tea production. In green tea, sucrose was metabolized faster by *Biovada* and *Prelude*, while xylose was used up only by *W. saturnus.* Methyl salicylate, as an aroma compound, was generated by 100 and 34 times in tea samples containing *W. saturnus* and *P. kluyveri*, respectively (Wang et al. 2020). In another study on tea non volatiles, caffeine was found to increase based on tea fermentation using *P. kluyveri* versus a decrease by *Prelude.* Fermented tea polyphenols and antioxidant ability were significantly enhanced by both *P. kluyveri* and *Biovada*, presenting an added value even though the underlying action mechanism has yet to be elucidated (Wang et al. 2020). Such novel contributions of previously known microbes need to be implemented for other strains that have been employed in other fermented products to assess their impact on tea metabolome.

The application of *Starmerella davenportii* Do18, a yeast strain isolated form kombucha beverage was tested in black tea fermentation. The study showed that it could grow at 1.5 pH, 37 °C, 40% sucrose, and 2% bile condition and survive both gastric and intestinal simulated conditions suggestive for its inclusion as potential probiotic in fermented tea products considering its stability. The strain could produce potential flavor compounds i.e., 2-phenylethanol (Tu et al. 2020) of desired rose-like odor presenting an added value of an improved aroma to the tea.

### Fungal cultures

The third contributing microorganism class in tea is fungi, of which several strains were detected in fermented tea samples (Wang et al. 2015). Such contribution is dominant in some cases, for instance, at early stages of Xiaguan Tuo tea fermentation with fungi cells up to 2.8 × 10^6^ per gram by two major species *Aspergillus niger* (*A*. *niger*) and *Blastobotrys adeninivorans* (*B*. *adeninivorans*) (Abe et al. 2008). Fungal growth depends on various factors, importantly moisture content, which explains the fluctuation of their growth during the fermentation stages. Upon availability of nutrients, temperature and humidity factors, fungi decompose and utilize carbon and nitrogen sources to release heat, which in turn raises the temperature that affects their growth. At a later stage, water content depletion occurs to negatively impact *A*. *niger* multiplication though found to be more suited for other microorganisms such as yeasts, which explains why yeasts dominate the late fermentation stages. Also, mold metabolic capacity to decompose cellulose and lignin (complex carbohydrates) generate mono- and oligo-saccharides that can be utilized by the yeasts that support their growth (Widlansky et al. 2005). Recently, the use of metagenomics enabled the identification of the glycoside hydrolase (GH) genes in *Aspergillus niger* as the core microbe responsible for pile-fermentation and aroma generation in Sichuan South-road Dark Tea(Zou et al. 2023).

In addition to *A. niger* and *B*. *adeninivorans*, *Penicillium* and *Rhizopus* were isolated. The antibacterial secretions from these organisms can contribute to tea safety. Reported studies revealed various activities for *B*. *adeninivorans* including enzyme secretion such as xylosidase, cellobiases, phytase, proteases, glucoamylase, among other activities that has led to the oxidization of polyphenols suggestive of their significant role in the transformation of raw tea structures (Li et al. 2018a).

*A*. *niger* was not only observed to be dominant in pu-erh tea, but rather involved in the development of aroma compounds. Such molecules are generated *via* microbial enzymatic activity within *A*. *niger* biosynthetic machinery. Likewise, as in Xiaguan Tuo tea, *A*. *niger* and *B*. *adeninivorans* were detected in pu-erh tea as the major fungal species. While monitoring tea conditions during fermentation process, it was observed that a temperature increase occurred at the start of the fermentation which stabilized around 50 °C on the 35th day followed by a decrease at the end of the fermentation period to reach a room temperature level. Such an increase in temperature is concurrent to a 30% loss of water content and a slight increase in acidity (5–6) pH throughout the fermentation. Such parameters are in favor of fungal growth during the fermentation period between day 10th and day 50th though associated with a decrease in polyphenols and inferring a link between fungal activity and the amount of bioactive compounds in tea (Abe et al. 2008). Whether novel biotransformed phenolics are produced in response to fungal enzymatic conversion has yet to be determined.

Fungal species and strains have significant influence on instant dark tea aroma. *Eurotium cristatum* and *A*. *niger* were found in Fu-brick tea produced by liquid-state fermentation, and to account for the generation of 100 volatiles. *Aspergillus* and *Eurotium*-based instant dark teas exhibit a woody and herbal aroma, while moldy aroma was present only in *Aspergillus*-based tea. Additionally, sequential inoculums of both teas produced sweet, minty, herbal and floral dark tea aroma (Chen et al. 2021), suggestive of the specificity of not only fungal type but order of addition in tea aroma development.

*E. cristatum* exhibits various health benefits to tea products such as inhibiting hyperglycemia or hyperlipidemia, reducing inflammation, lowering obesity, exhibiting antidysenteric activity, regulating gut microbiota, modulating dysbiosis, and relieving ulcerative colitis. Thus, it is considered as a potential probiotic that could add health values to fermented tea (Chen et al. 2021; Lu et al. 2022). How it functions in combination with other microbes during tea fermentation has yet to be investigated? In *E. cristatum*-based dark instant tea, more diversified metabolic pathways likely accounted for the high number of free aroma molecules. Glycoside precursors decomposed to provide energy upon utilizing carbohydrates required for fungal growth. Specifically, *A*. *niger* rapidly decomposed both cellulose and hemicellulose, providing the needed energy for fungal development (Chen et al. 2021).

Further, *A*. *niger* under optimum culture conditions could produce high content of theabroennins (TB) in instant dark tea. These bioactive pigments are produced by the effect of microbial polyphenol oxidase and peroxidase, which provide positive health effects in allergic response, atherosclerosis and antihyperlipidemia. The optimum conditions of ~ 27.5 mL/g liquid-solid ratio, 5.4% (v/v) *Aspergillus* inoculum, and 184 r/min rotation speed led to the production of 290 g/Kg TB (Wang et al. 2017; Huang et al. 2019). Whether these conditions are optimized for aroma compounds in tea has not yet been fully determined.

As a treatment drug for respiratory system disorders, theophylline can be produced from the microbial conversion of caffeine. However, during the production of green, black, white and oolong teas, certain organisms if present have the capability to convert caffeine to theophylline. For example, *Aspergillus sydowii* can effectively convert caffeine into theophylline during aerobic fermentation of pu-erh tea. Such conversion effect of 8% *A. sydowii* required a room incubation temperature, and 35% moisture content to generate ca. 25 mg/g theophylline from 30 mg/g caffeine (Zhou et al. 2019). However, other isolated species of *Aspergillus niger, Aspergillus pallidofulvus, Aspergillus sesamicola and Penicillium mangini* showed no effect on theophylline and enhanced caffeine level, suggestive that by monitoring of fungal consortium, changes in tea chemicals could be predicted to gurantee ultimate product quality. In that context, adding known microbial species or strains explored to produce certain chemicals to the tea fermentation process under optimized conditions should be considered if targeting certain bioactivity, reaction, or bioconversion yield.

Other species of *Aspergillus* isolated from pu-erh tea fermentation such as *Rhizomucor pusillus, Aspergillus tubingensis, Aspergillus fumigatus, Aspergillus marvanovae*, and *Rhizomucor tauricus* showed the ability to convert polyphenols into TB upon fermenting sun-dried green tea infusion. In terms of production method of TB and when compared to SSF in the case of *A. tubingensis*, yielded TB through the above-mentioned submerged pure culture fermentation demonstrated superiority as it had a higher yield (10 g/L) compared with the SSF method (3 g/L) (Wang et al. 2015). Such fermentation model represents an alternative, productive and controlled pathway if TB is the targeted tea product to be optimized.

Several fungi are though known as sources of mycotoxins such as aflatoxins. Thus, safety studies should be periodically conducted to ensure that their levels are below detection limits. For example, out of 30 microbial metabolites in pu-erh instant tea, none of them showed detecÒÒ quantities of aflatoxins B_1_, G_1_, B_2_, G_2_, cyclopiazonic acid, fumonisins B_1_, B_2_, B_3_ or ochratoxin A (Wang et al. 2019). More studies are required to investigate a wider range of possible toxins in various fermented teas with several microorganisms. Recently, Xu et al. reviewed the common mycotoxins and discussed sources of potential masked mycotoxins in dark tea (Xu et al. 2023).

Fuzhuan brick tea fermentation condition led to the isolation of *Eurotium, Aspergillus, Beauveria, Verticillium, Rhizomucor* and *Pestalotiopsis.* Interestingly, *Beauveria* was observed to exert protection against insect-related tea spoilage. As the dominant strain in this tea, *E. cristatum* colonies multiplied rapidly to reach 6 log CFU/g dry weight concurrent with the production of anthraquinones i.e., physcion and emodin, which have several biological activities likely derived from fungal metabolism (Xu et al. 2011) being absent from tea metabolome. Further studies are required to optimize to produce higher yield brick tea and identify other novel compounds derived from the fermentative fungi combined with quality evaluation and determination of its health benefits.

Another important fungus, *Aspergillus egyptiacus* was detected in dark fermented tea which is considered as marker for tea quality (Xu et al. 2018c). However, its metabolic pathway and resultant metabolites have not been fully investigated.

In a study conducted on pu-erh tea, high temperature and acidic parameters were observed as the optimal growing conditions for several fungi. The isolates were dominated by *Rasamsonia emersonii, R. pusillus, A. fumigatus* and *A. niger* in high numbers of 10^7^ and 10^6^ (*niger)* copies /g dry tea. The growing conditions favor thermophilic fungi (temperature of 50–65 °C, pH of 4.5-5.0 over most of the fermentation days, and moisture content of 45%). The microbial growth dynamics of different microbes during fermentation initially experienced dominance of aerobic microbes leading to oxygen drop and oxidation of some tea components such as organic acids resulting in acidic conditions that favor fungal growth. Also, the relatively lower numbers of *A. niger* was associated with its inability to grow at 55 °C. *A. niger* accounts for the development of crimson soup due to tannase production that catalyzes tannins hydrolysis. In contrast, pu-erh tea cholesterol lowering effect can be related to the presence of lovastain produced by *A. fumigatus* and another non dominant *A. tubingensis.* This controlled study could detect previously unrevealed *R. emersonii*, aerobically thermophilic fungi and amylolytic, hemicellulolytic, cellulolytic and pectinolytic enzymes producer. Other similar studies with modified methodology to tailor the growth of a wider range of microbes can lead to new isolates with potential functions that have not been detected in fermented tea (Zhang et al. 2016).

## Organoleptic characteristics of fermented teas

**Fig. 3:**
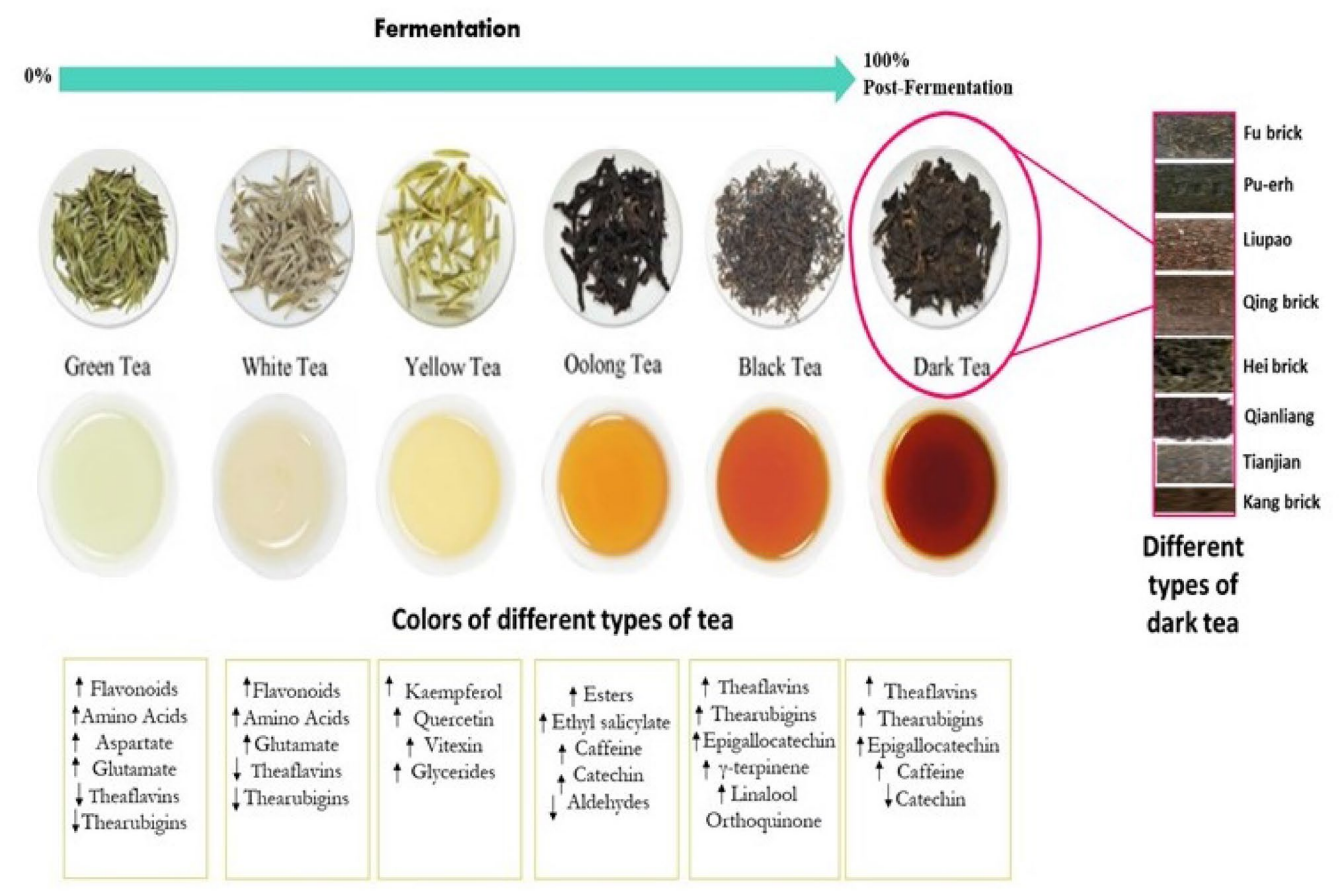
The color of branch and liquors of the different tea types

Compared to other tea types, white tea is devoid of fermentation stages, and only passes through decolorization and drying (Tan et al. 2017). Regarding green tea, chlorophyll proved to be the influential component for the color of dry tea leaves; water-insoluble chlorophylls were also released from the fragile tea leaves during infusion and increased both the greenness and turbidity of tea infusions (Mandal et al. 2022). Among flavonoids detected in green tea infusions, quercetin was shown to be the most important phenolic contributing for the greenness of tea infusion. The fresh leaf of variety yellow tea has a yellow color and is plucked from albino tea cvs. processed with the same processing technology used for green tea. It is produced by using a unique procedure known as “sealed yellowing”, in which green color of the fresh leaves turns into yellow (Xu et al. 2018a). Unlike green, black, and oolong tea, dark tea has unique color including black-auburn appearance and orange-red tea mixture (Fig. 3). Dark tea is, thus, gradually favored by consumers due to its unique sensory features and beneficial health effects. Microbial fermentation is the key factor responsible for such characteristics (Chen et al. 2018). Catechins begin to polymerize into larger molecules *via* condensation with the aid of oxidation, and non-volatile components such as TFs and TRs are produced and to account for the color of black tea (Pasrija et al. 2015). The green color of tea leaves transforms to coppery brown during the fermentation process. Theaflavins are responsible for the briskness, brightness, and quality of tea liquor, meanwhile, the taste, color, and body are more reflected by thearubigins level. It was also found that theaflavins and thearubigins are responsible for orange-red and reddish-brown color, respectively (Stodt et al. 2014). A relationship between liquor brightness and the chemical composition of black tea was also noted. A positive correlation is usually observed between liquor brightness and theaflavin of black tea (Mandal et al. 2022). The liquor brightness, both determined using chemical and sensory analyses, consistently declined with increasing fermentation time, irrespective of rise in TF levels such that the shorter the fermentation duration and the lower the fermentation temperature, the higher was the brightness.

Regarding tea aroma, the name of “white tea” originates from silky white feathers covering unripe leaves and buds as it has a delicate and sweet taste different from unrivaled flavor of green tea (Damiani et al. 2014). During decolorization step, level of amino acid found in tea increases, which were subsequently oxidized during the fermentation process. Levels of aspartic acid and glutamic acid are like those of green tea according to tea types, and other amino acids are most abundant in white tea among tea varieties (Horanni and Engelhardt 2013). The yellow tea has distinctive stale, aged, and fungal aroma; and mellow, sweet, and smooth taste with low levels of bitterness and astringency. Some volatile components are produced owing to the conversion of some aroma precursors, besides the formation of TFs and TRs during the fermentation process. Amino acids combined with orthoquinone, which is an oxidized structure of catechin, play the key role in deciding black tea aroma (Mandal et al. 2022). The cracked green leaves had a raw smell that subsided over time. As the fermentation prolongs, at a specific time, a fruity aroma develops which also diminishes over time, summarizing the first nose. More distinct fruity aroma appears when the coppery brown color forms, which is called the second nose. Once the second nose is noticed, the fermentation process is finished (Sharma and Rao 2009).

Astringency in black tea can be a tangy or non-tangy type. The former is characterized by a sharp and puckering action with little aftertaste, whereas the latter is characterized as tasteless, mouth drying, and mouth coating, with a lingering (more than 60 s) after taste. Decaffeination process may further result in the formation of non-tangy from tangy, altering the nature of astringency. Caffeine alongside black tea polyphenols is necessary for the expression of reasonable levels of tangy astringency (Chaturvedula and Prakash 2011; Sharma and Rao 2009). Both TFs and TRs derived from the oxidation of catechins and their gallates during the fermentation stage contribute to the taste of black tea brews/beverages (Asil et al. 2012). Briskness and/or astringency is usually associated with TFs and the unoxidized catechins, especially gallated catechins, epicatechin gallate (ECG) and epigallocatechin-3-gallate (EGCG) (Mandal et al. 2022). The taster’s evaluation of briskness of tea liquors showed that 20 °C fermentation temperature produced significantly brisker teas than 30 °C suggestive for the importance of temperature monitoring during the fermentation process. It was also shown that the processing conditions which favored less degradation of simple TFs, and the retention of higher residual ECG and EGCG levels produced brisker tea liquors. Maintaining a low fermentation temperature and short duration will ensure less conversion of ECG and EGCG but greater formation of the simple and dominant TF. The resultant black teas are then brisk, and bright and probably offer more benefit to human health.

### Conclusion and future directions

Recently, the continuous growth in consumers´ demand for fermented foods is attributed mainly to their myriad of health benefits and unique sensory characteristics. Fermented tea is considered as a convenient source of components that can function as health promoting, and nutritional support with improved qualities. Fermentation is the core process that requires orchestration of several organisms such as bacteria, yeast, and fungi under certain growth condition to ultimately yield the unique sensory or functional attributes in tea. One of the future promising areas of research is fermented tea development lies in its quality and safety. Such a goal can be achieved through optimization of fermentation variables targeting higher yield of metabolites which enhances sensory qualities to fit market preferences. Identification of new microorganisms, studying the relationship between the various growing variables, microbial communities and metabolites production is important for fermentation optimization. Moreover, identification of different recipes that can impart a key health promoting metabolites and exhibit novel flavor, taste, or color. Optimization of growth conditions to harvest high or low number of certain organisms can aid the fast-track production of fermented products. Optimization of initial nutrient composition as well as the metabolic profile of the constitution microorganisms can aid the determination of best reaction directions with higher metabolites yield. Metagenomics sequencing of these new microorganisms should aid reveal their biosynthetic machineries and biotransformation capacities prior to fermentation. Cross application of fermenting microorganisms from other products such as meat, milk, and cereals with their distinguished sensory features, health benefit and high safety in tea fermentation ought to be considered to aid in expediting such goal outcome. Applying bioinformatics tools can be a novel approach enhancing fermented tea as such database knowledge can ease searching about novel genomes of crucial molecules that can be produced in fermented tea product. In addition, advances in metabolomics techniques can enhance fermentation efficiency through profiling and monitoring of composition, biochemical changes, and holistically encompass envisaged metabolism which aid in adjusting the proper attributes and maximize outcomes. Controlling safety issues including presence of pathogenic microorganisms, toxic metabolites produced, or unclean production set need to be well addressed during tea fermentation. Cleaning validation studies need to be practiced on the machinery system used in the production, risk assessment of constituting microorganisms or generated metabolites with safety controls upon them should be implemented. Additional control of decontamination step and packaging preservation technologies need to be considered while producing a batch of fermented tea at a large scale to suit industrial level needs.

## Data Availability

All the data are included in the manuscript.
